# Effect of *Ocimum sanctum L* as LDD in periodontal therapy 

**DOI:** 10.6026/97320630019590

**Published:** 2023-05-31

**Authors:** Burra Anand Deepika, Jaiganesh Ramamurthy

**Affiliations:** 1 Department of Periodontics, Saveetha Dental College and Hospitals, Saveetha Institute of Medical and Technical Sciences, Saveetha University, Chennai, Tamil Nadu, India

**Keywords:** *Ocimum sanctum L* (Tulsi), Periodontitis, Tetracycline, Local drug delivery

## Abstract

*Ocimum sanctum L* (Tulsi) has various properties like antibacterial, anti-inflammatory and anti-oxidant. To compare the effect of the local-drug delivery system containing 2% *Ocimum sanctum L* (Tulsi) as an adjunct to scaling and root planing (SRP).The main aim of the study was to evaluate the efficacy of *Ocimum sanctum L* (Tulsi) gel with Tetracycline fibers (Actisite) for the treatment of periodontitis patients. 40 subjects with periodontitis (pocket depth of 5 mm) were selected and divided into 2 groups Group I: *Ocimum sanctum L* (Tulsi) gel (n= 20) and Group II: Tetracycline fibers (Actisite) (n = 20). Clinical parameters assessed were Gingival Index , Plaque Index , Probing Depth and Clinical Attachment Loss were assessed at baseline, 1 month, 3 months, 6 months, 8 months. Our results showed that Gingival index and Plaque index for for GROUP I: *Ocimum sanctum L* (Tulsi) and GROUP II: Tetracycline fibers (Actisite)are not statistically significant p>0.05 for baseline, at 1 month, 3 months, 6 months, 8 months. Probing depth and Clinical attachment are not significant p>0.05 for baseline, at 1 month, 3 months, 6 months, and statistically significant difference seen at 8 months p<0.05. 2% *Ocimum sanctum L* (Tulsi) gel can be effectively used as an adjunct to scaling and root planing. When used as an adjunct to scaling and root planing, it helps in reduction of pocket depth and gain of clinical attachment. *Ocimum sanctum L* (Tulsi) showed promising results when compared to Tetracycline fibers (Actisite).

## Background:

Periodontitis is a multifactorial disease that impairs the tooth's supporting structures. Chronic periodontitis, systemic disease-associated periodontitis, and necrotizing periodontitis are examples of periodontitis. Periodontal disease is also caused by a local bacterial infection with pathogenic microflora in the periodontal pocket. The inflammatory process is triggered by microbial plaque and bacterial infection [1]. Bacteria in the periodontal pocket produce a highly organised and intricate biofilm, which eventually spreads subgingivally and is challenging to remove during regular oral cleaning. Gram negative anaerobic bacteria make up the majority of the periodontal disease [2].

Antibacterial drugs have been used in conjunction with mechanical debridement to treat periodontal infection. Because of the limited access in the periodontal pocket, the efficacy of all methods is limited [3]. Due to the intricate structure of the root and the location of the lesion, traditional treatment methods like mechanical debridement which removes the subgingival flora and creates a clean, smooth, and biocompatible root surface might not always be effective. Controlling supragingival plaque is essential to prevent recolonization. It has been shown in numerous clinical studies that scaling and root planing along with proper oral hygiene causes a shift in the subgingival plaque, which is sufficient to stop periodontal disease in the majority of patients. Oral hygiene is essential for a successful course of treatment because patients who do not control their plaque adequately during or after therapy are more likely to develop recurrent periodontitis [4].

Antibacterial medications are used in conjunction with mechanical debridement to treat periodontal infections. The outcome is limited due to a lack of accessibility. Because the periodontal pocket provides an ideal environment for the growth of anaerobic pathogenic bacteria, antibiotics must reach the pocket's depth for effective treatment. The ideal requirements of the local drug delivery should be delivered to the bottom of the pocket via the drug delivery system. It should only be effective against periodontal infections, not the commensal microbiome. The medication must be antibacterial. The planned dose must be sufficient to kill the target organism with no side effects. It should have a long shelf life. It must be biodegradable and biocompatible. Plant extracts are now commonly utilised as the primary component in mouthwash to reduce gingival inflammation, and gel versions are commonly used to treat periodontitis [5].

*Ocimum sanctum L* (Tulsi) belongs to the basil family Lamiaceae. *Ocimum sanctum L* (Tulsi) is an aromatic shrub. *Ocimum sanctum L* (Tulsi) was shown to have many qualities which can manage the interplay between the microbes and the body’s immune response. The interaction between the host immune inflammatory mediators and pathogenic microorganisms denotes the development of the periodontitis. Effectiveness of *Ocimum sanctum L* (Tulsi) in many formulations was studied. In situ or topical application of Ocimum sanctum L (Tulsi) in the form of liquid extract and in gel forms were researched [6].

Tetracycline has been used for a very long time to treat periodontal disease. This is a typical treatment for aggressive localised periodontitis that is resistant to other treatments. Tetracycline-containing fibers are the first drug to be made locally available. Tetracycline is a bacteriostatic antibiotic that prevents bacterial protein synthesis and inhibits tissue collagenase activity. These are 0.5mm-diameter, 23cm-long threads of non-resorbable biologically inert plastic copolymers (ethylene and vinyl acetate) that are loaded with 25% weight-for-weight tetracycline HCL powder. When inserted into the periodontal pocket, it is well absorbed by oral tissues and keeps tetracycline concentrations stable for 10 days. Tetracycline fibres that are biodegradable have recently been developed and are sold as periodontal plus AB, which degrades in 7 days [7]. Our team has extensive knowledge and research experience that has translated into high quality publications [8- 9,10,11 12,13,14]. Therefore, it is of interest to determine the efficacy of *Ocimum sanctum L* (Tulsi) plant extract in comparison with Tetracycline fibers (Actisite) for the treatment of periodontitis patients by randomized clinical trial.

## Material and Methods:

## Preparation of 2% *Ocimum sanctum* gel and Supercritical fluid (SCF):

**Figure 1:**
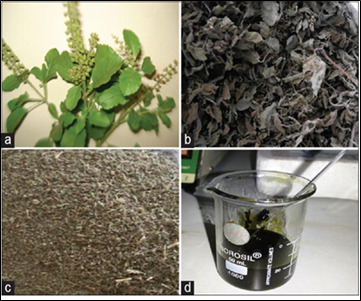
The image depicts a) *Ocimum sanctum L* (Tulsi) leaves; b) Dried *Ocimum sanctum L* (Tulsi) leaves; c) Powdered Ocimum sanctum L (Tulsi) d) *Ocimum sanctum L* (Tulsi) gel

**Figure 2:**
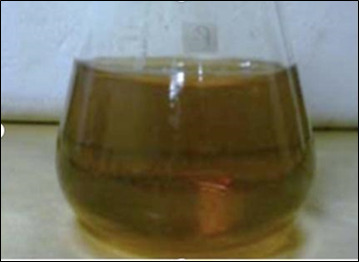
The image depicts the preparation of Supercritical fluid (SCF) - 250 grams of *Ocimum sanctum L* (Tulsi) powder.It is soaked in 1000 ml of Ethyl alcohol for 48 hours.

250 grams of *Ocimum sanctum L* (Tulsi) powder (Table 1) is taken and soaked in 1000 mL of Ethyl alcohol for 48 hours. It is filtered with Whartman's filter. Filter liquid is evaporated that is Supercritical Fluid (SCF). The SCF is stored in the fridge Figure 1 and Figure 2.

Preparation of 2% *ocimum sanctum* gel: (Refer Table 1)

## Preparation of *Ocimum sanctum L* (Tulsi) gel:

**Figure 3:**
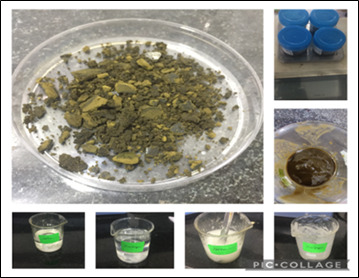
The Image depicts the *Ocimum sanctum L* (Tulsi) gel prepared using *Ocimum sanctum L* (Tulsi) leaf dried extract, Carbopol, HPMC. Prepared *Ocimum sanctum L* (Tulsi) gel is stored in air tight container boxes and stored in the fridge.

Carbopol 940 was submerged overnight in distilled water that contained 0.2% sodium benzoate. HPMC solution, Propylene glycol and 2 ml of SCF (Homogenized) were added. Triethanolamine was added in drops and checked for pH.The pH ranges from 6-6.5. At room temperature, the *Ocimum sanctum L* (Tulsi) gel was kept. For a period of six months, the *Ocimum sanctum L* (Tulsi) gel that has been made is firm. Changes in pH were documented and corrected in accordance with protocol Figure 3.

## Application of *Ocimum sanctum L* (tulsi) gel as LDD:

After phase I therapy, 2% *Ocimum sanctum L* (Tulsi) gel is in liquid form (under refrigeration) and was loaded into a 5ml syringe with a needle attached to it. With increasing temperature as in oral cavity, gel formulation occurred, which could be used as a local drug delivery, given to Group I subjects (n=20) are shown in to periodontal pocket and clinical parameters were assessed at baseline, 1 month, 3 months, 6 months and 8 months.

## Study design:

The study design include a Randomized controlled clinical trial for the subjects came from outpatient department of periodontics, Saveetha dental college and hospitals for the eligibility criteria for the study population are as follows:

## Inclusion criteria:

[1] Patients with generalized chronic gingivitis.

[2] Patients in the age group of 20-65 years.

[3] Systemically healthy subjects with Gingival index score, Plaque index score > 1 , Probing depth 5mm , Clinical attachment loss 5mm at the time of examination.

## Exclusion criteria:

[1] Patients with gingivitis

[2] Smokers

[3] Antibiotic therapy within last 6 months of the study

[4] Pregnant and lactating women

[5] Patients undergone or having undergone periodontal therapy within the last 6 months of study.

With the above Inclusion criteria and Exclusion criteria a total of 40 subjects were included in the study.

## Study group:

40 subjects with chronic localized or generalized periodontitis with pocket depth of 5 mm. 40 periodontitis subjects were divided into 2 groups and sites were randomly selected to group I and group II by tossing a coin.

Group I: *Ocimum sanctum L* (Tulsi) gel (n= 20)

Group II: Tetracycline fibers (Actisite) (n = 20)

*Ocimum sanctum L* (Tulsi) and Tetracycline fibers (Actisite) were given to subjects. Both groups received SRP (scaling and Root planing) and *Ocimum sanctum L* (Tulsi) gel (n= 20) was given to Group I and Tetracycline fibers (Actisite) was given to Group II. The assessment criteria included Gingival Index (GI) score, Plaque Index (PI) score, Probing Depth (PD) and Attachment Loss (AL) score that were assessed at baseline, 1 month, 3 months, 6 months, and 8 months.

## Statistical analysis:

Differences between the study groups were statistically analyzed by SPSS Software 23.0 version; PAIRED "T" TEST (Intra group) was done to analyse the difference between the groups. Inter group comparisons were analyzed by unpaired t test. Mean and Standard Deviation were assessed for statistical analysis and the results are tabulated. p< 0.05 was considered a significant difference.

## Result

A total of 40 sites in 40 subjects were treated. 20 sites received *Ocimum sanctum L* (Tulsi) as LDD and 20 sites received Tetracycline fibers (Actisite) as LDD. At the end of 1 month all sites were healed uneventfully and follow up was done up to 8 months are discussed in tables (Table 2, Table 3, Table 4,Table 5, Table 6, Table 7) neither complications nor allergic reactions that could be related to the *Ocimum sanctum L* (Tulsi) treatment modalities were also observed.

## Discussion:

Plant extracts are possible sources of novel antimicrobial components especially against bacterial microorganisms. An important feature of plant extracts and their constituents is hydrophobicity which makes them divide the lipids portion of the cell membrane of bacteria and mitochondria interrupting the structures of cells and making them more absorbent. Plants have different forms of bioactive compounds. It also has different forms of phytochemical compounds [15]. The antimicrobial activity of many plant extracts has been previously reviewed.

The present study was conducted to assess the efficacy of *Ocimum sanctum L* (Tulsi) gel compared to Tetracycline fibers (Actisite) for the management of periodontal disease. *Ocimum sanctum L* (Tulsi) gel is effective demonstrating its potential use as efficient and in addition used as standard control for the management of periodontitis. *Ocimum sanctum L* (Tulsi) gel is used as mouthwash rinses and gel for the treatment of gingivitis and periodontitis. It is also used against oral microbes. Gupta et al. conducted a triple-blinded randomized controlled trial to test the efficacy of 4% w/v mouthrinse containing tulsi and 0.12% chlorhexidine. It was found that the mouthrinse containing O. sanctum was as effective in reducing gingivitis [16]. Deepika et al. concluded that 2% of *Ocimum sanctum L* (Tulsi) showed that it is effective in reducing gingival bleeding and gingival inflammation. It also helps in reducing the Plaque. *Ocimum sanctum L* (Tulsi) showed no side effects when compared to Chlorhexidine (CHX) [17]. Mallikarjuna et al. concluded that *Ocimum Sanctum* (Tulsi) at 5% and 10% concentrations showed better inhibition zones against *Aggregatibacter actinomycetemcomitans*. They showed smaller inhibition zones against Prevotella intermedia and Porphyromonas gingivalis. Hence proved that *Ocimum Sanctum* (Tulsi) can be utilized as an efficient adjunct and in addition to the regular periodontal treatment [18]. Ramamurthy *et al.* conducted a study about *Ocimum Sanctum* (Tulsi) gel that demonstrated possible anti-oxidant and anti-inflammatory effects. It is less toxic than brine shrimp nauplii. *Ocimum Sanctum* (Tulsi) proved to be the most favorable agent for the therapy of periodontal conditions [19]. There were no studies on *Ocimum sanctum L* (Tulsi) (in gel form) as a local drug-delivery system hence the study was planned to assess the efficacy of *Ocimum sanctum L* (Tulsi) gel compared to Tetracycline fibers (Actisite) for the management of periodontal disease. However Adriana et al. observed similar reductions in mean plaque index gingival index sulcus bleeding index probing pocket depth; and gain in clinical attachment level using 1% chlorhexidine gel as an adjunct to SRP [20]. In our study *Ocimum sanctum L* (Tulsi) was used as LDD in gel form we found that Our results showed that Gingival index and Plaque index for GROUP I: *Ocimum sanctum L* (Tulsi) and GROUP II: Tetracycline fibers (Actisite) are not statistically significant p>0.05 for baseline at 1 month 3 months 6 months 8 months. Probing depth and Clinical attachment loss for GROUP I: *Ocimum sanctum L* (Tulsi) and GROUP II: Tetracycline fibers (Actisite) are not significant p<0.05 for baseline at 1 month 3 months 6 months and statistically significant difference seen at 8 months p<0.05.

## Conclusion:

2% *Ocimum sanctum L* (Tulsi) gel can be effectively used as an adjunct to scaling and root planing. When used as an adjunct to scaling and root planing, it helps in reduction of pocket depth and gain of clinical attachment. It is a beneficial antimicrobial, anti-inflammatory and anti-plaque agent. It is biologically well accepted by the oral tissues and showed good acceptability with no side effects by all the subjects in the study. *Ocimum sanctum L* (Tulsi) can be used as a local drug-delivery system for the management of periodontal disease. However, long term studies are required to validate the results.

## Figures and Tables

**Table 1:** The table depicts the ingredients of preparation of 2% Ocimum sanctum. L (Tulsi) gel; HPMC – Hydroxy Propyl Methylcellulose; SCF - Super Critical Fluid

**INGREDIENTS**	**QUANTITY**
Carbopol 940	2g
Polymer (HPMC)	2g
Tulsi SCF extract	2ml
Sodium benzoate	0.2ml
Propylene glycol	5ml
Triethanolamine	q.s
Distilled water	q.s to make 100ml

**Table 2:** Gingival index

**Time interval**	**Group I: *Ocimum Sanctum*.L (Tulsi) Mean ± SD**	**Group II: Tetracycline fibers(Actisite) Mean ± SD**	**p value**
Baseline	2.710± 0.65	2.702± 0.12	0.214
1 month	2.614±0.50	2.582± 0.75	0.11
3 month	2.120± 0.45	1.981± 0.31	0.336
6 month	1.870± 0.68	1.527± 0.55	0.12
8 month	1.524±0.19	1.516± 0.31	0.102
The significance of statistical tests for gingival index for GROUP I: *Ocimum Sanctum*.L (Tulsi) and GROUP II: Tetracycline fibers (Actisite) are not significant P>0.05 for baseline, at 1 month, 3 months, 6 months, 8 months.

**Table 3:** Plaque index

**Time interval**	**Group I: *Ocimum Sanctum*.L (Tulsi) Mean ± SD**	**Group II: Tetracycline fibers(Actisite) Mean ± SD**	**p value**
Baseline	2.245 ± 0.45	2.252 ± 0.16	0.11
1 month	2.182 ± 0.10	2.024 ± 0.35	0.21
3 month	1.850 ± 0.25	1.426 ± 0.38	0.832
6 month	1.535 ± 0.62	1.342 ±_0.25	0.706
8 month	1.216 ± 0.14	1.124 ±0.38	0.211
The significance of statistical tests for plaque index for GROUP I: *Ocimum Sanctum*.L (Tulsi) and GROUP II: Tetracycline fibers (Actisite) are not significant P>0.05 for baseline, at 1 month, 3 months, 6 months, 8 months.

**Table 4:** Mean Probing depth in group I and group II patients

**Time interval**	**Group I: *Ocimum Sanctum*.L (Tulsi) Mean ± SD**	**Group II: Tetracycline fibers(Actisite) Mean ± SD**	**p value**
Baseline	5.81± 0.12	5.72 ± 0.16	0.211
1 month	5.24± 0.24	5.17 ± 0.32	0.226
3 month	4.82±_0.37	4.65 ± 0.26	0.822
6 month	4.43± 0.61	4.24 ± 0.31	0.618
8 month	4.12± 0.43	3.91 ± 0.51	0.002
The significance of statistical tests for Probing depth for GROUP I: *Ocimum Sanctum*.L (Tulsi) and GROUP II: Tetracycline fibers (Actisite) are not significant p>0.05 for baseline, at 1 month, 3 months, 6 months, and statistically significant difference seen at 8 months p<0.05.

**Table 5:** Mean Clinical Attachment Loss in group I and group II patients

**Time interval**	**Group I: *Ocimum Sanctum*.L (Tulsi)Mean ± SD**	**Group II: Tetracycline fibers(Actisite) Mean ± SD**	**p value**
Baseline	7.56 ± _0.37	7.45 ± _0.27	0.715
1 month	7.38 ± 0.55	7.18 ±0.42	0.611
3 month	6.68±0.61	6.34 ± 0.16	0.803
6 month	6.12±0.27	5.92 ± 0.63	0.794
8 month	5.28±0.34	4.89 ±0.27	0.015
The significance of statistical tests for clinical attachment loss for GROUP I: *Ocimum Sanctum*.L (Tulsi) and GROUP II: Tetracycline fibers (Actisite) are not significant p>0.05 for baseline, at 1 month, 3 months, 6 months, and statistically significant difference seen at 8 months p<0.05.

**Table 6:** Subjective Criteria Analysis Acceptability

**Subjects 30**	**Acceptable 30**	**Tolerance x**	**Intolerance x**
**Discomfort**
Subjects	Absent	Present	
30	30	x	
**Burning Sensation**
Subjects	Absent	Present	
30	30	x	
**Dryness/Soreness**
Subjects	Absent	Present	
30	30	x	

**Table 7:** Objective Criteria Analysis Ulcer formation

**Ulcer formation**
**Subjects**	**Absent**	**Present**
30	30	x
